# Comparing patient-reported outcomes measurement information system^®^ (PROMIS^®^)-16 domain scores with the PROMIS-29 and 5-item PROMIS cognitive function scores

**DOI:** 10.1007/s11136-024-03747-4

**Published:** 2024-08-14

**Authors:** Chengbo Zeng, Ron D. Hays, Anthony Rodriguez, Janel Hanmer, Patricia M. Herman, Maria Orlando Edelen

**Affiliations:** 1 Patient Reported Outcomes, Value and Experience (PROVE) Center, Department of Surgery, Brigham and Women’s Hospital, Boston, MA USA; 2 Division of General Internal Medicine and Health Services Research, UCLA Department of Medicine, Los Angeles, CA USA; 3 Behavioral and Policy Sciences, RAND Corporation, 20 Park Plaza #920, Boston, MA USA; 4 Division of General Internal Medicine, University of Pittsburgh, Pittsburgh, PA USA; 5 Behavioral and Policy Sciences, RAND Corporation, 1776 Main Street, Santa Monica, CA USA

**Keywords:** PROMIS-29, PROMIS-16, Domain, Psychometric evaluation

## Abstract

**Purpose:**

This study evaluates the interpretability of Patient-Reported Outcomes Measurement Information System^®^ (PROMIS^®^)-16 profile domain scores (physical function, ability to participate in social roles and activities, anxiety, depression, sleep disturbance, pain interference, cognitive function – abilities, and fatigue) compared to the PROMIS-29 scores and a 5-item PROMIS cognitive function score. The study aims to provide insights into using these measures in clinical and research settings.

**Methods:**

Analyses were conducted using data from 4130 adults from a nationally representative, probability-based internet panel between September and October 2022. A subset of 1256 individuals with back pain was followed up at six months. We compared the PROMIS-16 profile with the corresponding domain scores from the PROMIS-29 and a custom five-item cognitive function measure. We evaluated (1) reliability through inter-item correlations within each domain and (2) criterion validity by comparing PROMIS-16 profile with the corresponding longer PROMIS measures: (a) standardized mean differences in domain scores, (b) correlations, and (c) concordance of change (i.e., got worse, stayed the same, got better) among those with back pain from baseline to six months later using the reliable change index. We report the Kappa coefficient of agreement and the frequency and percentage of participants with concordant classifications.

**Results:**

Inter-item correlations for the PROMIS-16 domains ranged from 0.65 in cognitive function to 0.92 in pain interference. Standardized mean differences between PROMIS-16 and the scores for the corresponding longer PROMIS domains were minimal (< 0.2). Correlations among the corresponding domain scores ranged from 0.82 for sleep disturbance to 0.98 for pain interference. The percentage of concordance in change groups ranged from 63% for sleep disturbance to 88% for pain interference. Except for sleep disturbance, the change groups derived from the PROMIS-16 showed moderate to substantial agreement with scores estimated from the longer PROMIS measures (Kappa coefficients ≥ 0.41).

**Conclusion:**

The PROMIS-16 domain scores perform similarly to the longer PROMIS measures and can be interpreted in the same way. This similarity indicates that PROMIS-16 can be useful for research as a brief health-related quality-of-life profile measure.

## Abstract (plain language summary)

The Patient-Reported Outcomes Measurement Information System^®^ (PROMIS^®^)-16 Profile assesses eight health-related quality of life domains (physical function, ability to participate in social roles and activities, anxiety, depression, sleep disturbance, pain interference, cognitive function – abilities, and fatigue) using two items per domain. We evaluated the PROMIS-16 profile in a sample drawn from a nationally representative, probability-based internet panel. The study supports the reliability and criterion validity of the PROMIS-16, showing that the domain scores closely align with and have high concordance in change with the PROMIS-29 scores and a custom five-item cognitive function score. The PROMIS-16 has the potential to be a brief health-related quality-of-life profile measure in research and clinical settings.

## Introduction

Patient-reported outcome measures (PROMs) are used to assess functioning, well-being, and health-related quality of life (HRQOL) [1–3]. The Patient-Reported Outcomes Measurement Information System^®^ (PROMIS^®^) assesses many aspects of physical, mental and social health. A subset of domains is included in profile measures that are widely used in research because of their favorable psychometric properties and scores normed to the United States general population [4, 5]. The PROMIS profiles provide both specific, actionable domain scores and global physical and mental health summary scores [6, 7]. However, the shortest adult PROMIS profile measure contains 29 items (4 items from each of seven domains and a pain intensity item), which may be too burdensome for use in routine clinical practice and some research contexts due to challenges of integration into existing clinical workflow and representativeness of the respondents [8]. This highlights the need for a concise PROMIS profile with adequate psychometric properties and similar interpretation as longer measures.

The PROMIS-16 profile was developed using empirical evaluation of data in a sample of 5775 respondents and stakeholder input of 50 candidate PROMIS items and item pairs [9]. It includes two items each to assess physical function, ability to participate in social roles and activities, anxiety, depression, sleep disturbance, pain interference, cognitive function – abilities, and fatigue. Beyond its simplicity, the PROMIS-16 was intended to provide scores comparable to those in the PROMIS-29 profile and the PROMIS cognitive function domains. The selected item pair for each domain showed strong psychometric properties.

The developmental work showed comparable results for the PROMIS-16 and corresponding longer PROMIS measures. These findings were based on data from a non-representative US sample of Amazon’s Mechanical Turk members [9]. They did not evaluate similarities from a longitudinal perspective, especially for patients with specific health conditions. The overall goal of this study is to re-evaluate the domain scores of the PROMIS-16 profile using data from a sample drawn from a nationally representative, probability-based internet panel. We also evaluate the PROMIS-16 domain scores’ ability to detect individual changes over six months relative to the longer PROMIS measures among participants with back pain. We hypothesize that the PROMIS-16 domain scores will (1) show similar distributions to and strongly correlate with the corresponding domain scores from longer PROMIS measures and (2) demonstrate individual changes comparable to those of the longer measures.

## Methods

### Data source and study sample

We analyzed longitudinal data from a sample drawn from a nationally representative, probability-based internet panel. These data were collected from KnowledgePanel members between September and October 2022. The KnowledgePanel was established in 1999 by Knowledge Networks and is currently operated by Ipsos Public Affairs. It is a high-quality probability-based panel with over 55000 members recruited through an address-based sampling method that uses the latest delivery sequence file of the US Postal Service [10–12]. This approach enhances the representation of the overall population and is particularly effective in recruiting underrepresented groups, such as young adults and minorities. Most KnowledgePanel members have internet access and computers; for those who do not, Ipsos provides them with a device and access as needed so that the panel reflects the full spectrum of US adults.

The survey included questions about demographic and clinical characteristics and PROMIS items. The survey was made available to 7224 individuals randomly selected from the 55000 KnowledgePanel members for completion within 10 days. The sample size (*n* = 7224) was based on the national prevalence of people with chronic back pain and our target of 1500 respondents with back pain in the original study [13]. Ipsos conducted some quality control and cleaning, and we included two fake conditions (i.e., “Syndomitis” and “Checkalism”) within a list of chronic health conditions to identify careless or insincere respondents [13]. Respondents who reported having back pain at baseline were followed up at six months to understand the changes of PROs using the same PROMIS items. Participants who responded to the baseline survey were entered into a monthly sweepstake for prizes; those eligible for and completed an additional survey on back pain and a 6-month assessment received 5000 points for each redeemable for cash (about $5).

**Fig. A1:**
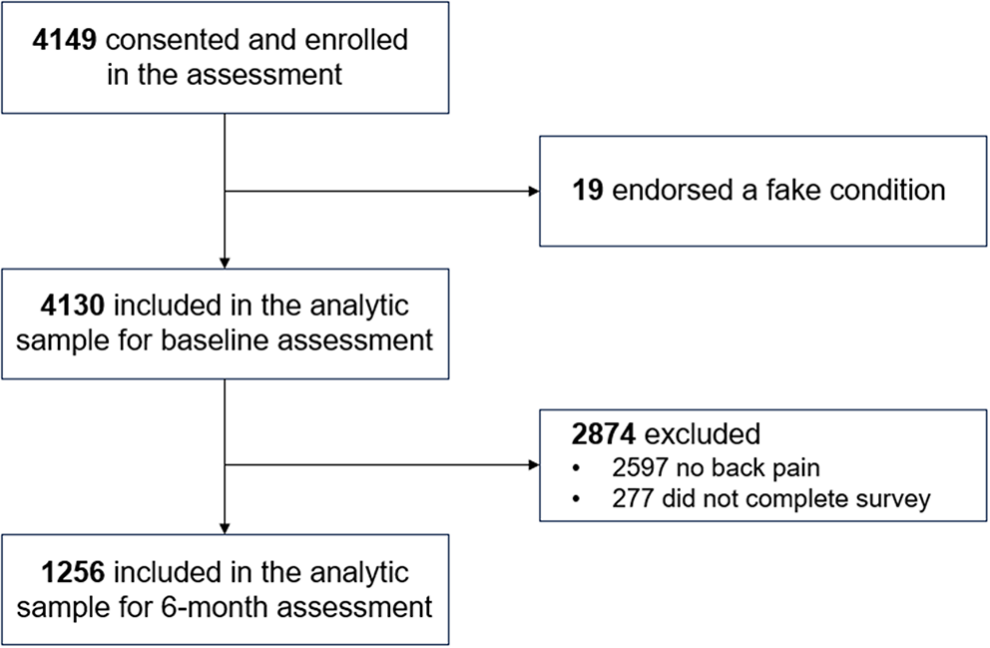
Study flowchart

Of the 4149 respondents (57%, 4149/7224) who consented and enrolled in the survey, 19 (0.5%) were excluded because they reported having a fake condition (i.e., “Syndomitis” and “Checkalism”) [13]. Differences in demographic characteristics between respondents and general populations can be adjusted using survey weights. However, given this study was a psychometric evaluation of the similarities between PROMIS-16 and longer PROMIS domain scores, minor differences in the demographic profiles of the sample and the general population would not change conclusions of the study. Thus, an unweighted sample was used. The final analytic sample for this study included 4130 participants at baseline, of whom 1533 (38%) reported having back pain and were assigned a follow-up survey at 6-months; 1256 (82% of the 1533 participants) completed the 6-month survey. Figure A1 in the appendix shows the flowchart of the analytic sample.

### Measures

#### PROMIS-16 profile

The PROMIS-16 profile measures eight domains (physical function, pain interference, fatigue, sleep disturbance, depression, anxiety, ability to participate in social roles and activities, and cognitive function-abilities) using two PROMIS items per domain. Many, but not all, of the PROMIS-16 items, are included in the PROMIS-29 + 2; five domains have both items, two have only one item, and one has no items from the PROMIS-29 + 2. Following the PROMIS convention and recommended scoring [9], we estimated IRT-based T-scores (mean = 50, SD = 10) for each PROMIS-16 domain.

#### Comparison with the PROMIS-29 and cognitive function domain scores

We compared the PROMIS-16 with the four-item domain scores from the PROMIS-29 + 2 profile for seven domains. For the domain of cognitive function – abilities, we created a five-item score by adding three additional items to the two contained in the PROMIS-29 + 2.

#### Demographic characteristics

We collected information on age, race/ethnicity, gender, education, and annual income.

### Statistical methods

We described the demographic characteristics of the sample using frequencies and percentages. We estimated polychoric correlations among items within each domain to evaluate internal consistency reliability. We classified correlation coefficients as weak (< 0.4), moderate (0.4–0.7), or strong (> 0.7) [14].

We evaluated the criterion validity of the PROMIS-16 profile by comparing it with the longer PROMIS measures (i.e., criterion measures) using (1) standardized mean differences (i.e., Cohen’s d) in domain scores [15], (2) correlations, and (3) concordance between individual changes. Since the five PROMIS-16 domains are the shortened versions of the longer PROMIS measures, and the other three domains share no more than one item with the longer measures, we hypothesize that there will be some minor differences in the domain scores. A Cohen’s d less than 0.2 was considered a trivial mean difference [15]. The correlations of corresponding domain scores between PROMIS-16 and the longer measures were used to evaluate convergent validity, with the hypothesis that the PROMIS-16 domain scores will be strongly correlated with those of the corresponding longer measures.

To evaluate the concordance of change groups between the PROMIS-16 and its corresponding longer PROMIS domains, we estimated the Reliable Change Index (RCI) for each domain in participants with back pain from baseline to the 6-month follow-up [16, 17]. Using a *p* < 0.05 cutoff of 1.96 on the RCI, we categorized participants into three change groups (got worse, stayed the same, got better). We evaluated the concordance of change groups using the frequency and percentage of participants with concordant classifications. We also calculated unweighted Kappa coefficients and 95% confidence intervals (CIs), and classified concordance as weak (≤ 0.2), fair (0.21–0.4), moderate (0.41–0.6), substantial (0.61–0.80), and strong (0.81–1.00) [18].

Given the small amount of missing data, we assumed that missingness was completely at random, and the analyses were conducted using complete cases. All analyses were performed using SAS version 9.4.

## Results

### Overview

Table 1 shows the demographic characteristics of the 4130 participants. Most (70%, *n* = 2887) were non-Hispanic White. Over half of the participants (59%, *n* = 2424) were 60 or younger. A total of 40% (*n* = 1667) of the participants had a bachelor’s degree or higher, and 73% had an annual income equal to or greater than $50,000. Those with back pain and who completed the 6-month assessment (30%, 1256/4130) tended to be older and have lower annual income than the rest of the sample (Table A1 in Appendix).

**Table 1:** Demographic characteristics of participants in KnowledgePanel sample (*N* = 4130)

Characteristics	*N* (%)
**Age**	
18 to 29 years of age	560 (13.6)
30 to 44 years of age	953 (23.1)
45 to 60 years of age	911 (22.1)
older than 60 years of age	1706 (41.3)
**Race/Ethnicity**	
White, non-Hispanic	2887 (69.9)
Black, non-Hispanic	414 (10)
Other, non-Hispanic	195 (4.7)
Multiracial, non-Hispanic	497 (12)
Hispanic	137 (3.3)
**Sex**	
Female	2035 (49.5)
Male	2049 (49.8)
Transgender	11 (0.3)
Do not identify as female, male, or transgender	17 (0.4)
**Education**	
No high school diploma or GED	279 (6.8)
High school graduate or GED	1097 (26.6)
Some college, or associate’s degree	1087 (26.3)
Bachelor’s degree	909 (22)
Master’s degree or higher	758 (18.4)
**Annual Income**	
Less than $10,000	121 (2.9)
$10,000 to 49,999	1006 (24.4)
$50,000 to 99,999	1258 (30.5)
$100,000 or more	1745 (42.3)

*Footnote* The sample sizes for some variables were smaller than the overall sample size because of missing data. GED: General education diploma

### Reliability

The inter-item polychoric correlations for the PROMIS-16 domains were high, indicating good internal consistency. Six out of the eight domains had inter-item correlations greater than 0.8. The domains of sleep disturbance (*r* = 0.67) and cognitive function abilities (*r* = 0.65) had inter-item correlations less than 0.8 but greater than 0.6.

### Criterion validity

#### Standardized mean differences in domain scores between PROMIS-16 and longer PROMIS criterion measures

Table 2 shows domain T-scores and standardized mean differences between the PROMIS-16 and longer PROMIS measures. The PROMIS-16 domain mean scores ranged from 47.2 (SD = 9.4) in the fatigue domain to 54.9 (SD = 8.3) in the ability to participate in social roles and activities domain, while the mean domain scores for the longer PROMIS measures ranged from 47.8 (SD = 9.8) in fatigue to 55.7 (SD = 8.9) in the ability to participate in social roles and activities. The standardized mean differences between the PROMIS-16 and the corresponding domain scores were minimal (< 0.2), ranging from − 0.12 in the cognitive function domain to 0.13 in the anxiety domain.

**Table 2:** Standardized mean differences in domain scores between PROMIS-16 profile and longer PROMIS measures

Domains	PROMIS-16	Longer PROMIS measures^†^	Cohen’s d
Physical function	50.7 (7.8)	50.6 (8.4)	0.02
Ability to participate in social roles and activities	54.9 (8.3)	55.7 (8.9)	-0.10
Anxiety	50.1 (8.7)	48.9 (9.1)	0.13
Depression	48.3 (8.7)	48.2 (9.0)	0.01
Sleep disturbance	48.8 (7.4)	48.3 (8.6)	0.06
Pain interference	50.0 (8.3)	49.3 (8.8)	0.08
Cognitive function, abilities	51.6 (8.8)	52.7 (9.9)	-0.12
Fatigue	47.2 (9.4)	47.8 (9.8)	-0.06

*Footnote* †: For cognitive function – abilities, a domain score based on the five items was used as the reference score; for the rest of the seven domains, PROMIS-29 domain scores were used as the references

#### Correlations of domain scores estimated by the PROMIS-16 and longer PROMIS criterion measures

The PROMIS-16 domain scores consistently showed strong correlations with those for the longer PROMIS measures, ranging from 0.82 for the sleep disturbance domain to 0.98 for the pain interference domain.

### Concordance of significant individual change in domain scores between PROMIS-16 and longer PROMIS criterion measures

The percentage of concordance in individual change ranged from 63% (789/1249) for the sleep disturbance domain to 88% (1100/1243) for the pain interference domain (Table 3). Seven out of the eight domains showed *moderate* to *substantial* agreement (Kappa coefficients ≥ 0.5). However, despite a 63% concordance rate of change groups in the sleep disturbance domain between PROMIS-16 and the longer PROMIS measures, the agreement statistic was only fair, with a Kappa coefficient of 0.29 (95%CI: 0.24–0.34).

**Table 3:** Concordance of significant individual changes based on the reliable change index for PROMIS-16 and longer PROMIS domain scores

PROMIS-16 domains	Longer PROMIS domains^‖^ (*n* [%])			Total (*n* [%]^†^)	Concordance (*n* [%])	Kappa (95%CI)
	Got worse	Stayed the same	Got better			
**Physical function**						
Got worse	**140 (11.3)**	34 (2.7)	0 (0)	174 (14)	1038 (83.5)	0.61 (0.56, 0.66)
Stayed the same	91 (7.3)	**813 (65.4)**	49 (3.9)	953 (76.7)		
Got better	0 (0)	31 (2.5)	**85 (6.8)**	116 (9.3)		
Total (n [%]^‡^)	231 (18.6)	878 (70.6)	134 (10.8)	1243 (100)		
**Ability to participate in social roles and activities**						
Got worse	**187 (15.0)**	38 (3.1)	0 (0)	225 (18.1)	967 (77.8)	0.61 (0.57, 0.65)
Stayed the same	97 (7.8)	**598 (48.1)**	83 (6.7)	778 (62.6)		
Got better	1 (0.1)	57 (4.6)	**182 (14.6)**	240 (19.3)		
Total (n [%]^‡^)	285 (22.9)	693 (55.8)	265 (21.3)	1243 (100)		
**Anxiety**						
Got worse	**166 (13.3)**	56 (4.5)	0 (0)	222 (17.8)	984 (80)	0.61 (0.57, 0.65)
Stayed the same	87 (7)	**649 (52.1)**	73 (5.9)	809 (64.9)		
Got better	0 (0)	46 (3.7)	**169 (13.6)**	215 (17.3)		
Total (n [%]^‡^)	253 (20.3)	751 (60.3)	242 (19.4)	1246 (100)		
**Depression**						
Got worse	**173 (14)**	26 (2.1)	0 (0)	199 (16.1)	1081 (87.4)	0.75 (0.71, 0.78)
Stayed the same	57 (4.6)	**745 (60.2)**	50 (4)	852 (68.8)		
Got better	0 (0)	24 (1.9)	**163 (13.2)**	187 (15.1)		
Total (n [%]^‡^)	230 (18.6)	795 (64.2)	213 (17.2)	1238 (100)		
**Sleep disturbance**						
Got worse	**99 (7.9)**	89 (7.1)	2 (0.2)	190 (15.2)	789 (63.2)	0.29 (0.24, 0.34)
Stayed the same	119 (9.5)	**581 (46.5)**	151 (12.1)	851 (68.1)		
Got better	6 (0.5)	93 (7.5)	**109 (8.7)**	208 (16.7)		
Total (n [%]^‡^)	224 (17.9)	763 (61.1)	262 (21)	1249 (100)		
**Pain interference**						
Got worse	**234 (18.8)**	26 (2.1)	0 (0)	260 (20.9)	1100 (88.5)	0.80 (0.77, 0.83)
Stayed the same	49 (3.9)	**643 (51.7)**	43 (3.5)	735 (59.1)		
Got better	0 (0)	25 (2)	**223 (17.9)**	248 (20)		
Total (n [%]^‡^)	283 (22.8)	694 (55.8)	266 (21.4)	1243 (100)		
**Cognitive function**,** abilities**						
Got worse	**162 (13)**	54 (4.3)	5 (0.4)	221 (17.8)	940 (75.5)	0.56 (0.51, 0.60)
Stayed the same	105 (8)	**614 (49.3)**	108 (8.7)	827 (66.4)		
Got better	2 (0.2)	31 (2.5)	**164 (13.2)**	197 (15.8)		
Total (n [%]^‡^)	269 (21.6)	699 (56.1)	277 (22.3)	1245 (100)		
**Fatigue**						
Got worse	**220 (17.6)**	80 (6.4)	0 (0)	300 (24)	958 (76.6)	0.61 (0.57, 0.65)
Stayed the same	90 (7.2)	**538 (43)**	53 (4.2)	681 (54.4)		
Got better	1 (0.1)	70 (6.6)	**200 (16)**	271 (21.7)		
Total (n [%]^‡^)	311 (24.8)	688 (55)	253 (20.2)	1252 (100)		

*Footnote* ‖: PROMIS-29 domain scores or PROMIS cognitive function score; †: column percentage; ‡: row percentage. CI: confidence interval. Cells in bold are those with consistent classification of change. The sample sizes for some variables were smaller than the overall sample size because of missing data

We found that more participants were classified as “stayed the same” in the PROMIS-16 profile compared to the longer PROMIS measures. For example, 77% of participants were in the “stayed the same” group for the PROMIS-16 versus 71% for the PROMIS-29 physical function domains (Table 3).

## Discussion

Using data from a nationally representative, probability-based panel, we evaluated PROMIS-16 domain scores in cross-sectional and longitudinal analyses. The results indicate small standardized mean differences and strong correlations between the corresponding PROMIS-16 and longer PROMIS domain scores, supporting their similarities. The longitudinal analyses of individual changes in participants with back pain from baseline to 6-month follow-up found moderate to substantial agreement in changes, except for sleep disturbance.

Fifty PROMIS items were considered for inclusion in the PROMIS-16 profile, and most of the selected PROMIS-16 items were derived from the PROMIS-29 + 2 profile which has been widely applied in clinical practice and research [6]. In addition to a comprehensive empirical evaluation of the 50 candidate items and their item pairs, the development process included collecting item preference ratings from stakeholders and an online sample from Amazon’s Mechanical Turk. This approach ensured that the selected items for each domain showed sound psychometric properties statistically and optimally reflected the respective domain from the perspectives of stakeholders and respondents [9]. Consequently, the PROMIS-16 profile is a measure that not only reflects the PROMIS-29 + 2 but also reduces the overall burden of data collection, making it feasible and easy to incorporate into research surveys and existing clinical workflows.

Our findings further corroborated the similarities between PROMIS-16 profile domain scores and the criterion measures. In cross-sectional analyses among all participants, the results demonstrated that the PROMIS-16 domain scores closely align with the corresponding criterion measure domains. In longitudinal analyses among participants with chronic back pain, we observed high concordance and moderate to substantial agreement in classifying changes between PROMIS-16 and corresponding criterion measure domains. These findings indicate that although the PROMIS-16 profile contains only two items per domain, it performs similarly to the longer PROMIS measures and can be interpreted similarly for the general population and potentially for those with specific health conditions. This evidence supports using the PROMIS-16 in clinical practice and research when clinicians seek a measure with relatively lower burden but comparable properties to existing longer measures.

Interestingly, sleep disturbance and cognitive function reflected the corresponding domains from longer PROMIS measures less accurately and demonstrated lower inter-item correlations than the other six domains. The possible reasons for this discrepancy could be due to the selected items and the longer PROMIS measures used for comparison. In the sleep disturbance domain, neither of the two selected items are included in the PROMIS-29 [9]. Consequently, using the domain score of sleep disturbance from the PROMIS-29 as a reference resulted in a relatively larger mean difference and lower agreement in individual change compared to other domains that shared at least one item with longer PROMIS measures. Further, the fact that the response options for the two sleep disturbance items in the PROMIS-16 were different likely reduced the magnitude of their inter-item correlation. In the cognitive function domain, although one of the two selected items was derived from the PROMIS-29 + 2, the criterion measure domain was constructed using five items, resulting in a relatively larger standardized mean difference for this domain [6, 19–21]. However, we found moderate agreement between cognitive function and its five-item criterion measure domain in change groups.

The study’s limitations warrant discussion. First, while the baseline sample was drawn from a panel nationally representative of US adults, only participants who reported back pain were surveyed at the 6-month follow-up. Therefore, caution is needed when interpreting the results of longitudinal analyses. Second, although about one-third reported back pain, future studies are encouraged to replicate our analyses in a clinical sample and in patients before and after a specific clinical event.

Our findings suggest that the domain scores of the PROMIS-16 profile generally reflect those of longer PROMIS measures, such as the PROMIS-29 domains plus a 5-item cognitive function domain scores. The PROMIS-16 profile can generate psychometrically sound (reliable and valid) domain-specific HRQOL scores. Future research is encouraged to evaluate these domain scores in different clinical contexts.

## Appendix

**See** Table A1 and Fig. A1.

**Table A1:** Demographic characteristics of participants with back pain and 6-month follow-up assessment (*N* = 1256)

Characteristics	*N* (%)
**Age**	
18 to 29 years of age	130 (10.4)
30 to 44 years of age	251 (20)
45 to 60 years of age	289 (23)
older than 60 years of age	586 (46.7)
**Race/Ethnicity**	
White, non-Hispanic	932 (74.2)
Black, non-Hispanic	98 (7.8)
Other, non-Hispanic	47 (3.7)
Multiracial, non-Hispanic	126 (10)
Hispanic	53 (4.2)
**Sex**	
Female	661 (52.7)
Male	589 (47)
Transgender	4 (0.3)
Do not identify as female, male, or transgender	0 (0)
**Education**	
No high school diploma or GED	87 (6.9)
High school graduate or GED	354 (28.2)
Some college or associate degree	364 (29)
Bachelor’s degree	245 (19.5)
Master’s degree or higher	206 (16.4)
**Annual Income**	
Less than $10,000	51 (4.1)
$10,000 to 49,999	351 (28)
$50,000 to 99,999	398 (31.7)
$100,000 or more	456 (36.3)

***Footnote*** The sample sizes for some variables were smaller than the overall sample size because of missing data. GED: General education diploma
